# Fatty acid signatures distinguish autotrophy, partial mycoheterotrophy, and full mycoheterotrophy in angiosperms

**DOI:** 10.1007/s00572-026-01303-1

**Published:** 2026-08-29

**Authors:** Kenji Suetsugu, Takahiro Kimoto, Ayano Medo

**Affiliations:** 1 Department of Biology, Graduate School of Science, Kobe University, 1-1 Rokkodai, Nada-ku, Kobe, Hyogo 657-8501 Japan; 2 Institute for Advanced Research, Kobe University, 1-1 Rokkodai, Nada-ku, Kobe, Hyogo 657-8501 Japan; 3 Department of Biology, Faculty of Science, Kobe University, 1-1 Rokkodai, Nada-ku, Kobe, Hyogo 657-8501 Japan; 4 Center for Ecological Research, Kyoto University, 2-509-3 Hirano, Otsu, Shiga 520-2113 Japan; 5 Faculty of Environmental Earth Science, Hokkaido University, N10W5, Kita-ku, Sapporo, Hokkaido 060-0810 Japan

**Keywords:** Arbuscular mycorrhiza, Fatty acid analysis, Fungal carbon acquisition, Mycoheterotrophy, Plant–fungus interactions, Rhizoctonia

## Abstract

Detecting partial mycoheterotrophy in green plants remains challenging, particularly in associations with arbuscular mycorrhizal (AM) or rhizoctonia fungi, because natural-abundance stable isotope patterns often provide limited diagnostic resolution in these systems. We tested whether fatty acid composition, widely used to infer trophic relationships in animal and soil ecology, provides complementary evidence of fungal carbon acquisition by plants. We characterized fatty acid profiles of 21 autotrophic reference plants, six partially mycoheterotrophic plants, and 17 fully mycoheterotrophic plants associated with AM, ectomycorrhizal, or saprotrophic fungi. In expanded analyses, we additionally examined three individuals each of the rhizoctonia-associated orchid *Goodyera biflora* and the presumed autotrophic AM plant* Trillium camschatcense* with marked ^13^C enrichment, together with six co-occurring autotrophic reference plants. Fatty acid composition differed significantly among the three nutritional groups. Increasing mycoheterotrophic dependence was associated with decreasing relative abundance of 18:3n-3 and increasing relative abundance of 18:2n-6, which is widely used as a fungal biomarker in soil ecology. Partially mycoheterotrophic plants occupied an intermediate position in ordination space, and all pairwise group comparisons were significant. Differentiation remained significant after exclusion of 18:3n-3 and 16:3n-3 and renormalization of the remaining components, indicating that chloroplast-associated fatty acids alone did not drive the pattern. *Goodyera biflora* resembled partially mycoheterotrophic orchids, whereas *T. camschatcense* resembled autotrophic references. Fatty acid profiling may therefore complement stable isotope analysis in assessments of partial mycoheterotrophy. The profiles likely reflect metabolic remodeling, incorporation of fungal-derived carbon into newly synthesized plant lipids, or both, although some retention of intact fungal lipids cannot be excluded.

**Supplementary Information:**

The online version contains supplementary material available at https://doi.org/10.1007/s00572-026-01303-1.

## Introduction

The “wood wide web” concept, which proposes that carbon, other resources, and signals can move among plants through common mycorrhizal networks, has attracted considerable public attention (Simard et al. 1997; Karst et al. 2023; Robinson et al. 2024). Although the frequency and ecological significance of such transfers remain debated, fungus-to-plant carbon transfer is well established in fully mycoheterotrophic plants (Merckx et al. 2024). Establishing how widespread partial mycoheterotrophy is among green plants is therefore important for understanding the ecology and evolution of mycorrhizal symbioses (Karst et al. 2023; Robinson et al. 2024; Merckx et al. 2024). Laboratory and in situ CO_2_-labeling experiments can trace short-term carbon movement among plants and fungi (Simard et al. 1997; Klein et al. 2016), but they are labor-intensive, technically demanding, and largely restricted to short-term fluxes (Hynson et al. 2013). Moreover, such experiments do not always distinguish transfer through common mycorrhizal networks from alternative pathways, including reassimilation of respired CO_2_ and uptake of root exudates (Karst et al. 2023; Robinson et al. 2024).

Consequently, natural-abundance stable isotope analyses, particularly of δ^2^H, δ^13^C, and δ^15^N, have become the principal approach for inferring fungal-derived nutrition in situ, especially in plants associated with ectomycorrhizal (ECM) or saprotrophic (SAP) fungi (Gebauer and Meyer 2003; Ogura-Tsujita et al. 2009; Hynson et al. 2013; Suetsugu et al. 2020). These inferences rely on the frequent, although not universal, enrichment of fungal tissues in ^2^H, ^13^C, and ^15^N relative to autotrophic plants (Gebauer and Dietrich 1993; Gleixner et al. 1993; Ziegler 1995; Hobbie et al. 1999; Griffith 2004). Among these isotopes, δ^13^C remains the principal indicator of fungal contributions to plant carbon budgets because it most directly reflects differences in carbon sources (Selosse et al. 2025).

Detecting partial mycoheterotrophy nevertheless remains difficult, particularly in green plants associated with arbuscular mycorrhizal (AM) or rhizoctonia fungi. In AM and rhizoctonia-associated systems, δ^13^C contrasts between putative mycoheterotrophs and autotrophs are often weak (Courty et al. 2011; Zahn et al. 2023). Furthermore, at least in AM plants, enrichment in ^2^H, ^13^C, or ^15^N can result from multiple known or potentially unrecognized physiological and environmental processes and thus does not uniquely indicate fungal-derived nutrition (Murata-Kato et al. 2022; Baan et al. 2025; Selosse et al. 2025). Accordingly, although enrichment in ^2^H and ^15^N has been proposed as complementary evidence (Hynson et al. 2013; Gebauer et al. 2016), a recent synthesis concluded that evidence for partial mycoheterotrophy in AM plants is substantial but remains indirect (Merckx et al. 2026).

These limitations highlight the need for complementary chemical approaches that provide evidence independent of bulk stable isotope patterns. Fatty acids are widely used as trophic markers in animal and soil food-web studies (Dalsgaard et al. 2003; Ruess and Chamberlain 2010; Jardine et al. 2020). Their utility derives from the taxonomically biased distributions of certain fatty acids and their relatively conservative transfer from dietary resources to consumers (Dalsgaard et al. 2003; Galloway and Budge 2020). In soil ecology, 18:2n-6 is commonly used as a broad fungal biomarker, whereas 16:1n-5 is widely used as an indicator of AM fungal biomass (Frostegård and Bååth 1996; Olsson 1999; Pollierer et al. 2010; Ngosong et al. 2012; Tsunoda et al. 2017; Lekberg et al. 2022).

Mycoheterotrophy can be viewed as a consumer–resource interaction because plants obtain organic nutrients directly from their fungal partners (Merckx 2013). Enrichment of bulk ^13^C and ^15^N in mycoheterotrophic plants relative to their fungal partners often parallels the trophic enrichment of consumers relative to their diets (Schiebold et al. 2017; Suetsugu et al. 2025; Suetsugu and Okada 2025b; but see also Gomes et al. 2023; Zahn et al. 2023). This conceptual parallel suggests that fungal-derived carbon may leave characteristic imprints on plant fatty acid profiles. Recent work further indicates that compound-specific δ^15^N analysis of amino acids, a method widely used in food-web studies (e.g., Ishikawa 2018), may help detect acquisition of fungal-derived organic nutrients by green plants (Suetsugu et al. 2026).

However, fatty acids are neither passive nor direct tracers of dietary sources because consumers can synthesize them *de novo* and selectively modify, retain, or metabolize them (Galloway and Budge 2020; Jardine et al. 2020). Many green plants transfer lipids to AM fungi, which depend on host-derived fatty acids (Jiang et al. 2017; Luginbuehl et al. 2017), and orchids may also transfer fatty acids to fungal partners during symbiotic germination (Zhao et al. 2025; Wang et al. 2026). Consequently, differences in fatty acid composition between mycoheterotrophic and autotrophic plants likely reflect some combination of retained fungal lipids, bidirectional carbon and nutrient exchange, metabolic remodeling associated with fungal carbon assimilation, and physiological variation in nutrient demand. Nevertheless, these composite profiles may remain informative about the degree of mycoheterotrophy, as they do in animal trophic ecology (Galloway and Budge 2020; Jardine et al. 2020).

Here, we tested whether fatty acid composition distinguishes among autotrophy, partial mycoheterotrophy, and full mycoheterotrophy in angiosperms. First, we asked whether overall fatty acid composition differed among these three nutritional strategies. Because chloroplast-associated fatty acids, particularly 16:3n-3 and 18:3n-3, predominate in photosynthetic taxa (Mongrand et al. 1998; He et al. 2020), these compounds could account for much of any observed differentiation. We therefore repeated the analyses after excluding both fatty acids. Finally, we assessed whether the fatty acid profiles of the rhizoctonia-associated orchid *Goodyera biflora* and the presumed autotrophic Paris-type AM plant* Trillium camschatcense* with marked ^13^C enrichment, more closely resembled those of autotrophic, partially mycoheterotrophic, or fully mycoheterotrophic plants.

## Materials and methods

### Study species and sampling localities

**Fig. 1:**
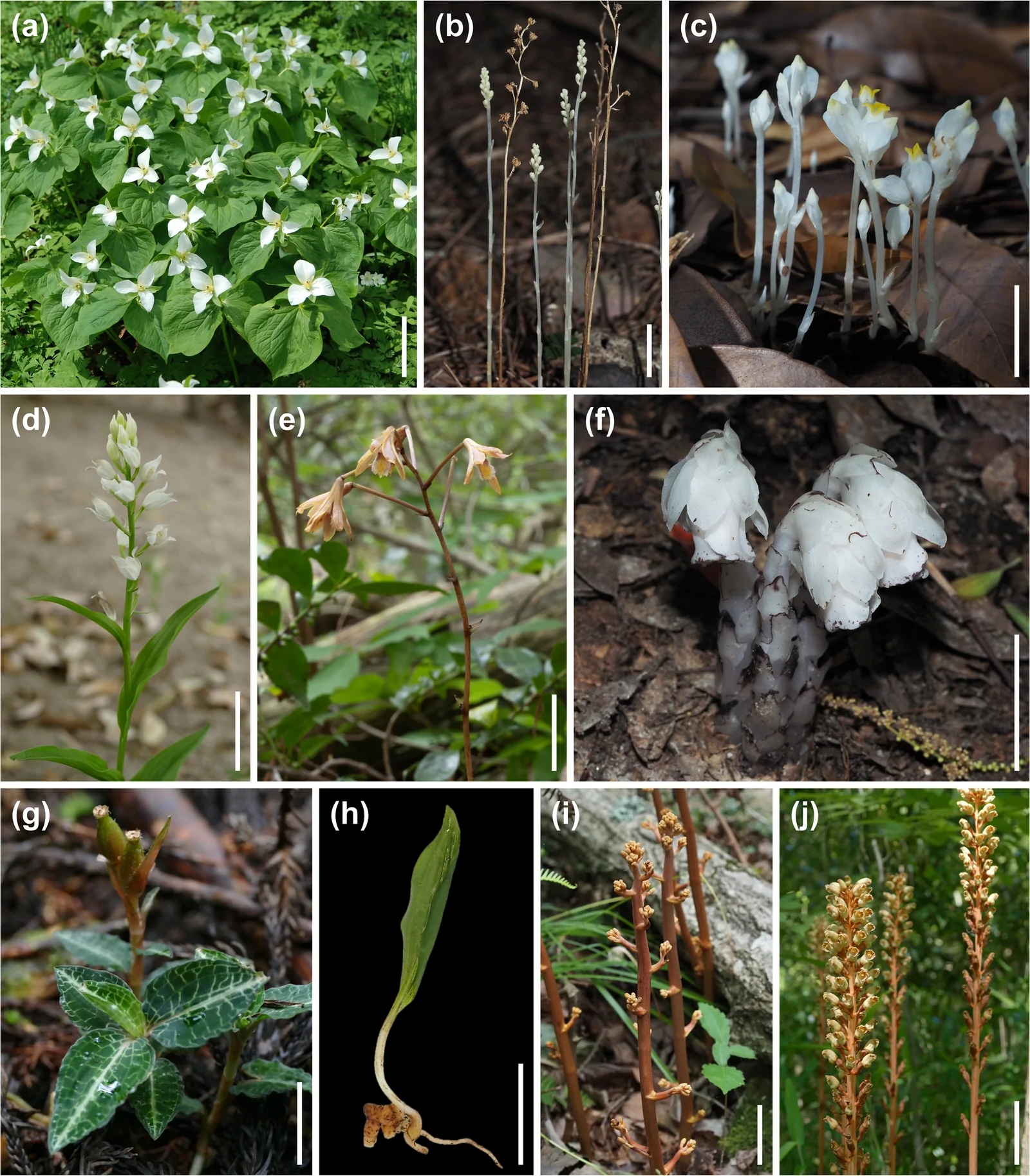
Target species analyzed in this study. (**a**) *Trillium camschatcense*, a presumed autotrophic species showing marked ^13^C enrichment. (**b**, **c**) Fully mycoheterotrophic plants associated with arbuscular mycorrhizal fungi: *Petrosavia sakuraii* (**b**) and *Burmannia cryptopetala* (**c**). (**d**) *Cephalanthera longifolia*, a partially mycoheterotrophic orchid associated with ectomycorrhizal fungi. (**e**, **f**) Fully mycoheterotrophic plants associated with ectomycorrhizal fungi: *Lecanorchis hokurikuensis* (**e**) and *Monotropastrum humile* (**f**). (**g**) *Goodyera biflora*, a rhizoctonia-associated orchid. (**h**) *Cremastra appendiculata*, a partially mycoheterotrophic orchid associated with wood-decaying fungi. (**i**, **j**) Fully mycoheterotrophic plants associated with wood-decaying fungi: *Cyrtosia septentrionalis* (**i**) and *Gastrodia elata* (**j**). Scale bars: 10 cm (**a**), 3 cm (**b**, **d–f**, **h**), 1 cm (**c**, **g**), and 5 cm (**i**, **j**)

At 10 sites spanning warm-temperate to cool-temperate regions of Japan, we sampled six fully mycoheterotrophic species associated with three fungal guilds—AM, ECM, and SAP—along with co-occurring autotrophic reference plants (Table S1). The fully mycoheterotrophic species comprised the AM-associated *Burmannia cryptopetala* and *Petrosavia sakuraii*, the ECM-associated *Lecanorchis hokurikuensis* and *Monotropastrum humile*, and the SAP-associated *Cyrtosia septentrionalis* and *Gastrodia elata* (*n* = 3 per species, except *G. elata*, for which *n* = 2; Fig. 1).

We also sampled two partially mycoheterotrophic orchids associated with ECM and SAP fungi, respectively (*Cephalanthera longifolia* and *Cremastra appendiculata*), and two focal green species: the rhizoctonia-associated orchid *Goodyera biflora* and the presumed autotrophic AM plant *Trillium camschatcense* with marked ^13^C enrichment (*n* = 3 per species; Murata-Kato et al. 2022). Individuals of *C. appendiculata* bearing coralloid rhizomes associate with wood-decaying Psathyrellaceae and depend strongly on fungal carbon, whereas those without coralloid rhizomes associate with rhizoctonia fungi and are predominantly autotrophic (Yagame et al. 2021; Zahn et al. 2022; Suetsugu et al. 2022; Suetsugu and Okada 2025a). We therefore included only individuals bearing coralloid rhizomes.

Co-occurring autotrophic reference species were collected within 2 × 2 m quadrats containing the target plants, with up to three reference species sampled per quadrat. We treated this reference group as operationally autotrophic because low-level fungal contributions to plant carbon budgets cannot be excluded for some AM-associated species, including *Oxalis* taxa proposed as candidates for partial mycoheterotrophy (Giesemann et al. 2021; Merckx et al. 2026). Following established protocols, we sampled understory reference plants at heights comparable to those of the target mycoheterotrophs (5–30 cm above ground) (Gebauer and Meyer 2003). Leaves were collected from photosynthetic species, including partially mycoheterotrophic taxa. For fully mycoheterotrophic taxa, we analyzed both scale leaves and floral stalks because scale leaves alone yielded insufficient material for fatty acid analysis. Samples were placed in a cooler immediately after collection and transported to the laboratory, where they were thoroughly rinsed with distilled water. Microscopic examination of sections from the sampled aboveground organs revealed no fungal structures; the remaining tissue was stored at − 20 °C within 24 h. Analyses restricted to species with well-supported nutritional classifications included 21 autotrophic reference samples from the corresponding sites. For the expanded analyses including *G. biflora* and *T. camschatcense*, we added six autotrophic reference samples from their sites, yielding 27 autotrophic reference samples in total.

## Fatty acid analysis

All samples were freeze-dried (FDM-1000, Tokyo Rikakikai Co., Ltd., Tokyo, Japan) for 12–48 h and finely cut with scissors and homogenized using an agate mortar and pestle. Lipids were extracted from 20 to 30 mg of dry tissue using the Bligh and Dyer method (Bligh and Dyer 1959). Each sample was sequentially mixed with 0.8 mL of distilled water, 2 mL of methanol, and 1 mL of chloroform, vortexed after each addition, and allowed to stand for 10 min. An additional 1 mL of chloroform and 1 mL of distilled water were added, and the mixture was centrifuged at 1,700 × g for 10 min. The lower chloroform-rich phase was collected, and the remaining material was extracted twice more with 2 mL of chloroform; after each extraction, the mixture was centrifuged at 1,700 × g for 10 min. The three chloroform-rich fractions were combined and evaporated to dryness under a stream of nitrogen at 37 °C. Using a modified procedure based on Ichihara and Fukubayashi (2010), the extracted lipids were hydrolyzed and methylated with 1 mL of a commercial hydrogen chloride–methanol reagent containing 5.0–10.0% HCl (w/w; product X0041; Tokyo Chemical Industry, Tokyo, Japan) at 95 °C for 3 h. After the addition of 2 mL of distilled water, the resulting fatty acid methyl esters (FAMEs) were extracted twice with 4 mL of hexane. After each extraction, the samples were vortexed and centrifuged at 1,500 × *g* for 5 min, and the upper hexane-rich phase was collected. The two hexane-rich fractions were combined, evaporated to dryness under a stream of nitrogen, and redissolved in 500 µL of hexane in a GC vial. Immediately before gas chromatography–mass spectrometry (GC–MS) analysis, 10 µL of methyl nonadecanoate (19:0) in hexane (100 µg mL^− 1^; GL Sciences Inc., Tokyo, Japan) was added to each vial as a retention-time reference.

FAMEs were analyzed using a 5977B GC/MSD system (Agilent Technologies, Santa Clara, CA, USA) equipped with a Select FAME GC capillary column (CP7420; 100 m × 0.25 mm i.d., 0.25 μm film thickness; Agilent). The initial column temperature was held at 140 °C for 2.5 min, increased to 240 °C at 4 °C min^− 1^, and then held at 240 °C for 15 min, resulting in a total run time of 42.5 min. Samples were injected in split mode at a split ratio of 5:1, with helium as the carrier gas. FAMEs were identified by comparing their retention times with those of a commercial standard mixture (37-component FAME mix, 47885-U; Supelco, Bellefonte, PA, USA) and their mass spectra with those in the NIST17.LIB and NIST21.LIB libraries (National Institute of Standards and Technology, Gaithersburg, MD, USA). We note that low-abundance fatty acids may have remained undetected when their peaks were obscured by non-target peaks.

Fatty acid nomenclature followed the conventional format X:Yn-Z, where X denotes the number of carbon atoms, Y the number of double bonds, and Z the position of the first double bond counted from the methyl end (e.g., 22:6n-3). The prefixes i- and a- denote iso- and anteiso-branched fatty acids, respectively. When the n-position could not be determined and distinction among isomers was unnecessary, FAMEs were reported without an n-position designation (e.g., 19:1). When unresolved isomers within the same carbon class could nevertheless be distinguished by their mass spectral fragmentation patterns and retention times, they were designated in order of retention using numerical suffixes after x (e.g., 16:1n-x1 and 16:1n-x2). Fatty acid composition was expressed as the percentage contribution of each peak to the summed peak area of all detected FAMEs, excluding the added 19:0 retention-time reference.

### Statistical analysis

All statistical analyses were conducted in R version 4.5.2 (R Core Team 2025), with statistical significance set at 0.05. Variation in fatty acid composition was visualized using nonmetric multidimensional scaling (NMDS) of Bray–Curtis dissimilarities. Ordinations were generated with metaMDS in vegan (Oksanen et al. 2025), using two dimensions (*k* = 2), a maximum of 100 random starts (trymax = 100), and no automatic data transformation (autotransform = FALSE). Fatty acids associated with ordination structure were identified by fitting vectors to the NMDS configuration with envfit in vegan; vectors were retained when permutation tests yielded *P* < 0.01. Differences in fatty acid composition were tested by permutational multivariate analysis of variance (PERMANOVA) based on Bray–Curtis dissimilarities, implemented with adonis2 in vegan. Pairwise PERMANOVAs were conducted with 9,999 permutations using pairwiseAdonis version 0.4.1 (Martinez Arbizu 2020). *P* values were adjusted using the Benjamini–Hochberg false discovery rate procedure. Similarity percentage (SIMPER) analysis, implemented with simper in vegan, identified the fatty acids contributing most to between-group dissimilarities. For the 18:2n-6/18:3n-3 ratio, the ratio was calculated for each sample and then averaged within each group.

NMDS, overall and pairwise PERMANOVA, and SIMPER analyses were conducted both before and after exclusion of the chloroplast-associated fatty acids 18:3n-3 and 16:3n-3. After exclusion, the relative abundances of the remaining fatty acids were rescaled to sum to 100%. Each analysis was applied to both the dataset restricted to species with well-supported nutritional classifications and the expanded dataset that included *Goodyera biflora*, *Trillium camschatcense*, and their co-occurring autotrophic reference plants.

## Results

Among species with well-supported nutritional classifications, fatty acid composition differed among autotrophic, partially mycoheterotrophic, and fully mycoheterotrophic plants. The largest contrasts involved 18:3n-3 and 18:2n-6. Relative to autotrophs (*n* = 21), fully mycoheterotrophic plants (*n* = 17) had substantially lower proportions of 18:3n-3 (4.0% ± 1.5% vs. 42.6% ± 6.8%; mean ± SD) and higher proportions of 18:2n-6 (45.8% ± 4.9% vs. 16.4% ± 5.1%). Partially mycoheterotrophic plants (*n* = 6) also differed from autotrophs, with higher proportions of 18:2n-6 (28.7% ± 4.2% vs. 16.4% ± 5.1%) and lower proportions of 18:3n-3 (31.6% ± 5.2% vs. 42.6% ± 6.8%) (Table 1; Table S1). The C_16_ monounsaturated fatty acid isomers with unresolved double-bond positions, 16:1n-x1 and 16:1n-x2, which may include the AM fungal biomarker 16:1n-5, were not detected in any AM-associated fully mycoheterotrophic sample.

**Table 1:** Mean (± SD) relative FAME composition in the expanded dataset by study group (% of summed endogenous FAME peak area)

Fatty acid	Study group				
	Autotroph (*n* = 27)	*T. camschatcense* (*n* = 3)	*G. biflora* (*n* = 3)	PMH (*n* = 6)	FMH (*n* = 17)
*Straight-chain saturated FAs*					
12:0	0.09 ± 0.12	0.27 ± 0.07	0.17 ± 0.15	0.07 ± 0.06	0.27 ± 0.36
14:0	0.58 ± 0.40	0.51 ± 0.08	0.28 ± 0.07	0.35 ± 0.24	0.61 ± 0.49
15:0	0.11 ± 0.05	0.15 ± 0.05	0.30 ± 0.05	2.04 ± 0.70	1.98 ± 3.02
16:0	20.30 ± 3.79	19.51 ± 0.77	20.12 ± 0.65	18.65 ± 0.40	25.93 ± 1.71
17:0	0.29 ± 0.16	0.31 ± 0.09	0.45 ± 0.09	1.11 ± 0.19	0.85 ± 0.49
18:0	2.58 ± 1.58	2.13 ± 0.20	2.64 ± 0.53	2.59 ± 0.51	3.14 ± 1.53
20:0	0.70 ± 0.88	0.52 ± 0.06	0.28 ± 0.24	0.55 ± 0.39	1.09 ± 1.20
21:0	0.05 ± 0.09	0.03 ± 0.00	0.05 ± 0.09	0.10 ± 0.11	0.19 ± 0.17
22:0	0.72 ± 0.60	0.95 ± 0.16	0.60 ± 0.13	1.49 ± 0.29	1.78 ± 1.20
23:0	0.20 ± 0.10	0.07 ± 0.01	0.37 ± 0.05	0.34 ± 0.08	0.91 ± 0.41
24:0	1.07 ± 0.80	1.47 ± 0.13	1.10 ± 0.27	1.26 ± 0.28	2.99 ± 1.58
25:0	0.12 ± 0.10	0.08 ± 0.02	0.07 ± 0.07	0.11 ± 0.04	0.34 ± 0.16
26:0	0.69 ± 0.86	0.14 ± 0.01	0.30 ± 0.48	0.32 ± 0.18	0.85 ± 0.70
27:0	N.D.	N.D.	N.D.	N.D.	0.03 ± 0.06
28:0	0.93 ± 1.20	0.19 ± 0.06	N.D.	0.41 ± 0.27	1.25 ± 1.58
30:0	0.81 ± 1.28	0.22 ± 0.05	N.D.	0.35 ± 0.44	0.20 ± 0.29
*Straight-chain monounsaturated FAs*					
16:1n-9	1.67 ± 0.65	2.27 ± 0.36	0.25 ± 0.03	0.14 ± 0.04	0.16 ± 0.05
16:1n-7	0.20 ± 0.11	0.07 ± 0.01	0.15 ± 0.04	0.55 ± 0.57	0.26 ± 0.26
16:1n-x1	0.03 ± 0.11	N.D.	N.D.	0.10 ± 0.05	0.10 ± 0.20
16:1n-x2	0.00 ± 0.01	N.D.	N.D.	N.D.	N.D.
17:1n-x1	0.00 ± 0.01	N.D.	N.D.	N.D.	0.03 ± 0.08
17:1n-x2	0.03 ± 0.04	0.06 ± 0.02	N.D.	0.04 ± 0.05	N.D.
18:1n-9	5.50 ± 4.72	2.65 ± 0.65	5.70 ± 1.68	6.87 ± 2.92	1.99 ± 1.33
18:1n-7	0.48 ± 0.32	0.24 ± 0.01	0.46 ± 0.09	1.05 ± 0.66	1.59 ± 0.80
18:1n-x1	0.01 ± 0.03	N.D.	N.D.	N.D.	N.D.
18:1n-x2	0.02 ± 0.09	0.02 ± 0.01	N.D.	0.14 ± 0.04	0.11 ± 0.25
18:1n-x3	0.00 ± 0.01	N.D.	N.D.	N.D.	N.D.
19:1	0.00 ± 0.02	N.D.	N.D.	N.D.	N.D.
20:1n-x	N.D.	N.D.	0.04 ± 0.03	N.D.	N.D.
20:1n-9	0.14 ± 0.16	0.71 ± 0.10	0.06 ± 0.06	0.13 ± 0.13	0.23 ± 0.22
22:1n-9	0.22 ± 0.27	0.12 ± 0.03	0.04 ± 0.04	0.05 ± 0.01	0.67 ± 0.72
24:1n-9	0.00 ± 0.02	N.D.	N.D.	N.D.	N.D.
*Straight-chain polyunsaturated FAs*					
16:2n-6	0.13 ± 0.28	N.D.	N.D.	N.D.	N.D.
16:2n-x	N.D.	N.D.	N.D.	0.09 ± 0.11	N.D.
16:3n-3	1.40 ± 3.32	N.D.	N.D.	N.D.	N.D.
18:2n-6	17.37 ± 5.03	20.00 ± 0.81	36.23 ± 2.96	28.68 ± 4.17	45.77 ± 4.87
18:2n-x1	0.00 ± 0.00	N.D.	N.D.	0.01 ± 0.02	N.D.
18:2n-x2	0.01 ± 0.02	N.D.	N.D.	0.02 ± 0.04	N.D.
18:3n-6	0.21 ± 0.07	0.31 ± 0.01	0.15 ± 0.08	0.11 ± 0.03	N.D.
18:3n-3	42.17 ± 6.25	46.15 ± 1.76	29.42 ± 1.75	31.63 ± 5.19	3.97 ± 1.46
20:2n-6	0.10 ± 0.08	0.19 ± 0.03	0.14 ± 0.04	0.05 ± 0.06	0.18 ± 0.09
20:3n-6	0.00 ± 0.02	N.D.	N.D.	N.D.	N.D.
20:3n-3	0.12 ± 0.07	0.27 ± 0.04	0.07 ± 0.06	0.06 ± 0.02	N.D.
20:4n-6	0.10 ± 0.54	N.D.	N.D.	N.D.	N.D.
20:5n-3	0.01 ± 0.05	N.D.	N.D.	N.D.	N.D.
22:2n-6	0.00 ± 0.01	N.D.	N.D.	N.D.	N.D.
*Branched-chain FAs*					
i-15:0	N.D.	N.D.	N.D.	N.D.	0.00 ± 0.02
a-15:0	N.D.	N.D.	N.D.	N.D.	0.02 ± 0.05
i-16:0	0.01 ± 0.02	N.D.	N.D.	N.D.	0.04 ± 0.09
a-16:0	N.D.	N.D.	N.D.	N.D.	0.05 ± 0.12
a-17:0	0.00 ± 0.03	N.D.	N.D.	N.D.	0.62 ± 1.39
i-18:0	0.02 ± 0.05	N.D.	N.D.	N.D.	0.05 ± 0.11
a-18:0	N.D.	N.D.	N.D.	N.D.	0.01 ± 0.03
a-19:0	N.D.	N.D.	N.D.	N.D.	0.04 ± 0.08
*Hydroxy FAs*					
16:0-OH	0.49 ± 0.34	0.25 ± 0.06	0.47 ± 0.10	0.47 ± 0.22	0.98 ± 0.42
18:0-OH	0.06 ± 0.20	N.D.	N.D.	N.D.	0.35 ± 0.48
20:0-OH	0.02 ± 0.06	N.D.	N.D.	N.D.	0.07 ± 0.08
24:0-OH	0.21 ± 0.23	0.15 ± 0.03	0.08 ± 0.07	0.06 ± 0.06	0.30 ± 0.24

PMH, partial mycoheterotroph; FMH, full mycoheterotroph; N.D., not detected in any sample within a category. Values displayed as 0.00 represent means below 0.005% after rounding

**Fig. 2:**
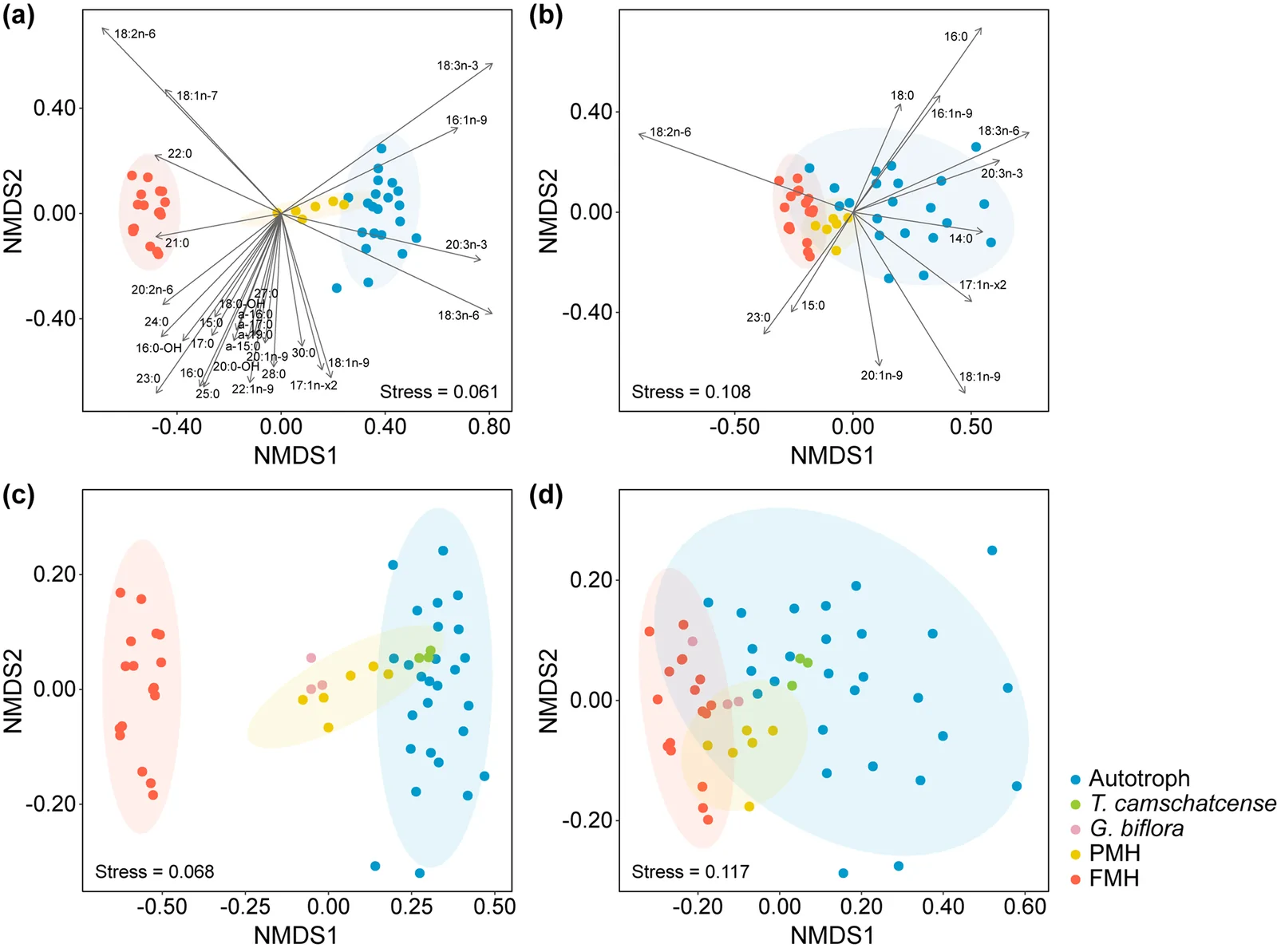
Nonmetric multidimensional scaling (NMDS) ordinations of fatty acid composition in autotrophic, partially mycoheterotrophic, and fully mycoheterotrophic plants, based on Bray–Curtis dissimilarities. Panels (**a**) and (**c**) show the complete fatty acid dataset, whereas panels (**b**) and (**d**) show the dataset after exclusion of the chloroplast-associated fatty acids 18:3n-3 and 16:3n-3. Panels (**a**) and (**b**) include only taxa with well-supported nutritional classifications; panels (**c**) and (**d**) additionally include *Goodyera biflora*, *Trillium camschatcense*, and their co-occurring autotrophic reference plants. Points represent individual samples, shaded regions indicate 95% confidence ellipses, and arrows in panels (**a**) and (**b**) indicate fatty acids significantly associated with the ordination (*P* < 0.01). PMH, partial mycoheterotroph; FMH, full mycoheterotroph

Autotrophic and fully mycoheterotrophic plants separated primarily along NMDS1. Autotrophic plants were associated with higher relative abundances of 18:3n-3, 16:1n-9, 20:3n-3, and 18:3n-6, whereas fully mycoheterotrophic plants were associated with higher relative abundances of 18:2n-6, 18:1n-7, 21:0, 22:0, and 20:2n-6 (Fig. 2a). Partially mycoheterotrophic plants occupied an intermediate position. PERMANOVA supported this pattern (Table S2), and all pairwise comparisons were significant (Table 2). Fully mycoheterotrophic plants differed from autotrophic plants (pseudo-*F* = 131.45, *R*^2^ = 0.785, *P* < 0.001) and from partially mycoheterotrophic plants (pseudo-*F* = 47.06, *R*^2^ = 0.691, *P* < 0.001). Autotrophic and partially mycoheterotrophic plants also differed, although less strongly (pseudo-*F* = 9.40, *R*^2^ = 0.273, *P* < 0.001). SIMPER identified 18:3n-3 and 18:2n-6 as the leading contributors. Together, they accounted for 67.9% of the dissimilarity between autotrophic and fully mycoheterotrophic plants; inclusion of 16:0 increased the cumulative contribution to 74.3% (Table S3). For autotrophic versus partially mycoheterotrophic plants, 18:2n-6 and 18:3n-3 again contributed most, together accounting for 52.1% of the dissimilarity; inclusion of 18:1n-9, 16:0, and 15:0 increased the cumulative contribution to 72.5% (Table S3).

**Table 2:** Pairwise PERMANOVA results for fatty acid composition across datasets

Dataset	Comparison	df	Sum of squares	pseudo-F	*R* ^2^	*P*
Base dataset with chloroplast-associated fatty acids	FMH vs. Autotroph	1	2.092	131.447	0.785	< 0.001
Base dataset with chloroplast-associated fatty acids	FMH vs. PMH	1	0.527	47.064	0.691	< 0.001
Base dataset with chloroplast-associated fatty acids	Autotroph vs. PMH	1	0.154	9.404	0.273	< 0.001
Base dataset without chloroplast-associated fatty acids	FMH vs. Autotroph	1	0.574	24.453	0.404	< 0.001
Base dataset without chloroplast-associated fatty acids	FMH vs. PMH	1	0.069	6.142	0.226	< 0.001
Base dataset without chloroplast-associated fatty acids	Autotroph vs. PMH	1	0.175	6.450	0.205	< 0.001
Expanded dataset with chloroplast-associated fatty acids	FMH vs. Autotroph	1	2.255	143.420	0.773	< 0.001
Expanded dataset with chloroplast-associated fatty acids	FMH vs. *T. camschatcense*	1	0.543	48.758	0.730	0.001
Expanded dataset with chloroplast-associated fatty acids	FMH vs. *G. biflora*	1	0.205	18.115	0.502	0.001
Expanded dataset with chloroplast-associated fatty acids	FMH vs. PMH	1	0.527	47.064	0.691	< 0.001
Expanded dataset with chloroplast-associated fatty acids	Autotroph vs. *T. camschatcense*	1	0.012	0.738	0.026	0.559
Expanded dataset with chloroplast-associated fatty acids	Autotroph vs. *G. biflora*	1	0.122	7.310	0.207	< 0.001
Expanded dataset with chloroplast-associated fatty acids	Autotroph vs. PMH	1	0.153	9.497	0.235	< 0.001
Expanded dataset with chloroplast-associated fatty acids	*T. camschatcense* vs. *G. biflora*	1	0.070	47.448	0.922	0.111
Expanded dataset with chloroplast-associated fatty acids	*T. camschatcense* vs. PMH	1	0.071	13.207	0.654	0.016
Expanded dataset with chloroplast-associated fatty acids	*G. biflora* vs. PMH	1	0.018	3.116	0.308	0.078
Expanded dataset without chloroplast-associated fatty acids	FMH vs. Autotroph	1	0.549	23.801	0.362	0.001
Expanded dataset without chloroplast-associated fatty acids	FMH vs. *T. camschatcense*	1	0.099	8.712	0.326	0.001
Expanded dataset without chloroplast-associated fatty acids	FMH vs. *G. biflora*	1	0.029	2.499	0.122	0.065
Expanded dataset without chloroplast-associated fatty acids	FMH vs. PMH	1	0.069	6.142	0.226	0.001
Expanded dataset without chloroplast-associated fatty acids	Autotroph vs. *T. camschatcense*	1	0.027	0.978	0.034	0.380
Expanded dataset without chloroplast-associated fatty acids	Autotroph vs. *G. biflora*	1	0.108	3.898	0.122	0.018
Expanded dataset without chloroplast-associated fatty acids	Autotroph vs. PMH	1	0.162	6.268	0.168	0.001
Expanded dataset without chloroplast-associated fatty acids	*T. camschatcense* vs. *G. biflora*	1	0.052	23.594	0.855	0.111
Expanded dataset without chloroplast-associated fatty acids	*T. camschatcense* vs. PMH	1	0.058	11.498	0.622	0.019
Expanded dataset without chloroplast-associated fatty acids	*G. biflora* vs. PMH	1	0.026	4.623	0.398	0.036
PMH, partial mycoheterotroph; FMH, full mycoheterotroph.						

The expanded dataset includes the base dataset plus *G. biflora*, *T. camschatcense*, and co-occurring autotrophic reference plants. *P* values were adjusted using the Benjamini–Hochberg false discovery rate procedure

After exclusion of the chloroplast-associated fatty acids 18:3n-3 and 16:3n-3, separation among autotrophic, partially mycoheterotrophic, and fully mycoheterotrophic plants weakened but remained significant. In the NMDS ordination, fully mycoheterotrophic plants remained associated with 18:2n-6 and saturated fatty acids such as 15:0 and 23:0, whereas autotrophic plants were associated mainly with 14:0, 16:0, 16:1n-9, 18:1n-9, 20:3n-3, and 18:3n-6 (Fig. 2b). Pairwise PERMANOVA confirmed that all contrasts remained significant (autotrophic vs. fully mycoheterotrophic plants: pseudo-*F* = 24.45, *R*^2^ = 0.404, *P* < 0.001; autotrophic vs. partially mycoheterotrophic plants: pseudo-*F* = 6.45, *R*^2^ = 0.205, *P* < 0.001; partially mycoheterotrophic vs. fully mycoheterotrophic plants: pseudo-*F* = 6.14, *R*^2^ = 0.226, *P* < 0.001; Table 2). In the SIMPER analysis, 18:2n-6 and 16:0 together accounted for 45.9% of the dissimilarity between autotrophic and fully mycoheterotrophic plants; inclusion of 18:1n-9 increased the cumulative contribution to 60.2% (Table S3). For autotrophic versus partially mycoheterotrophic plants, the principal contributors were 18:2n-6 (27.6%), 16:0 (16.9%), and 18:1n-9 (12.5%), which together accounted for 57.0% of the dissimilarity; inclusion of 15:0 and 16:1n-9 increased the cumulative contribution to 67.0% (Table S3).

In the expanded dataset including *G. biflora*, *T. camschatcense*, and their co-occurring reference plants, autotrophs (*n* = 27) had high relative proportions of 18:3n-3 (42.2% ± 6.3%) and low proportions of 18:2n-6 (17.4% ± 5.0%). Partially mycoheterotrophic plants (*n* = 6) and *G. biflora* (*n* = 3) had lower proportions of 18:3n-3 (31.6% ± 5.2% and 29.4% ± 1.8%, respectively) and higher proportions of 18:2n-6 (28.7% ± 4.2% and 36.2% ± 3.0%, respectively). By contrast, *T. camschatcense* (*n* = 3) more closely resembled autotrophic references, with a high proportion of 18:3n-3 (46.1% ± 1.8%) and a relatively low proportion of 18:2n-6 (20.0% ± 0.8%; Table 1). The mean individual 18:2n-6/18:3n-3 ratio showed the same pattern: values were low in autotrophs and *T. camschatcense* (0.42 and 0.43, respectively), intermediate in partially mycoheterotrophic plants and *G. biflora* (0.94 and 1.23), and highest in fully mycoheterotrophic plants (12.53).

In the NMDS ordination of the expanded dataset, *G. biflora* clustered near partially mycoheterotrophic plants, whereas *T. camschatcense* clustered with autotrophic plants (Fig. 2c). Pairwise PERMANOVA likewise showed that *T. camschatcense* did not differ from autotrophic plants (pseudo-*F* = 0.74, *R*^2^ = 0.026, *P* = 0.559) but differed from fully mycoheterotrophic plants (pseudo-*F* = 48.76, *R*^2^ = 0.730, *P* = 0.001) and partially mycoheterotrophic plants (pseudo-*F* = 13.21, *R*^2^ = 0.654, *P* = 0.016; Table 2). In contrast, *G. biflora* differed from autotrophic plants (pseudo-*F* = 7.31, *R*^2^ = 0.207, *P* < 0.001) and fully mycoheterotrophic plants (pseudo-*F* = 18.12, *R*^2^ = 0.502, *P* = 0.001), but not from partially mycoheterotrophic plants (pseudo-*F* = 3.12, *R*^2^ = 0.308, *P* = 0.078; Table 2). After exclusion of the chloroplast-associated fatty acids (Fig. 2d), *T. camschatcense* again did not differ from autotrophic plants (pseudo-*F* = 0.98, *R*^2^ = 0.034, *P* = 0.380) but differed from fully mycoheterotrophic plants (pseudo-*F* = 8.71, *R*^2^ = 0.326, *P* = 0.001) and partially mycoheterotrophic plants (pseudo-*F* = 11.50, *R*^2^ = 0.622, *P* = 0.019). *Goodyera biflora* remained distinct from autotrophic plants (pseudo-*F* = 3.90, *R*^2^ = 0.122, *P* = 0.018) and differed from partially mycoheterotrophic plants (pseudo-*F* = 4.62, *R*^2^ = 0.398, *P* = 0.036), but not from fully mycoheterotrophic plants (pseudo-*F* = 2.50, *R*^2^ = 0.122, *P* = 0.065; Table 2).

## Discussion

Fatty acid composition differed clearly among autotrophic, partially mycoheterotrophic, and fully mycoheterotrophic plants. Organ identity likely contributed to some of this variation because leaves were analyzed for autotrophic and partially mycoheterotrophic taxa, whereas scale leaves and floral stalks were analyzed for fully mycoheterotrophic taxa. Differences in chloroplast- and cuticle-associated lipids may therefore have influenced the observed profiles. Nevertheless, autotrophic and partially mycoheterotrophic plants were both represented by leaves, yet their fatty acid composition differed significantly. This within-organ contrast, together with the consistently intermediate position of partially mycoheterotrophic plants in both the NMDS ordination and the relative abundances of major fatty acids, indicates that organ identity alone cannot explain the observed differentiation. Much of the pattern was associated with a progressive decline in chloroplast-associated fatty acids, particularly 18:3n-3, as fungal dependence increased (Whitaker 1986; Reszczyńska and Hanaka 2020; Yu et al. 2021).

In the complete fatty acid profiles, 18:2n-6 accounted for approximately 17% of total FAMEs in autotrophs, 29% in partially mycoheterotrophic plants, and 46% in fully mycoheterotrophic plants. Importantly, differentiation among the groups persisted after exclusion of the two chloroplast-associated fatty acids and renormalization of the remaining components. Consistent with this result, 18:2n-6 was significantly associated with the fully mycoheterotrophic side of the NMDS ordination both before and after exclusion of 18:3n-3 and 16:3n-3. A higher relative proportion of 18:2n-6 combined with a lower relative proportion of 18:3n-3 may therefore provide a useful compositional signal of increasing fungal dependence. This pattern is notable because 18:2n-6 is widely used as a fungal biomarker in soil ecology (Kaiser et al. 2010; Ruess and Chamberlain 2010; Kühn et al. 2019). Similarly, 18:1n-7 contributed to the separation of fully mycoheterotrophic plants and may reflect broader microbial inputs because it is abundant in both AM fungi and some bacteria (Olsson 1999; Olsson and Johansen 2000; Quideau et al. 2016). Together, these patterns raise the possibility that fungal-derived compounds contribute to the fatty acid pools of mycoheterotrophic plants.

However, 18:2n-6 is also a common constituent of plant lipids and can occur at high relative abundance in photosynthetic tissues (Whitaker 1986; Mongrand et al. 1998; He et al. 2020); its abundance alone therefore cannot demonstrate direct incorporation of fungal lipids. No fungal structures were observed in microscopic sections of the sampled aboveground organs, so the fatty acid profiles should not be interpreted as direct detection of fungal biomass in these tissues. Moreover, the relative abundance of 18:1n-9, which is sometimes used as a fungal biomarker in soil studies (Kaiser et al. 2010), did not increase consistently with mycoheterotrophy. More importantly, C_16_ monounsaturated isomers potentially including 16:1n-5, a widely used marker of AM fungal biomass (Olsson 1999; Lekberg et al. 2022), were not detected in any AM-associated fully mycoheterotrophic sample. Their absence, together with the inconsistent enrichment of other proposed fungal markers, suggests that the observed profiles are unlikely to reflect simple accumulation of intact fungal lipids and may instead largely reflect plant metabolic remodeling associated with reduced or lost photosynthetic function.

The lack of recognizable signatures of intact fungal lipids does not exclude the contribution of fungal-derived carbon to plant lipid metabolism. Fatty acids may undergo differential uptake, turnover, transport, and metabolic processing. In fully mycoheterotrophic plants, fungal carbon transfer can occur through digestion of fungal hyphae (Merckx 2013; Bougoure et al. 2014), and the resulting compounds may be metabolized belowground before their carbon is incorporated into de novo synthesized plant fatty acids (Zhao et al. 2024). Thus, fungal carbon may enter plant lipid pools without preserving the diagnostic fatty acid composition of its source. These possibilities could be tested by supplying fungi with ^13^C-labeled fatty acids or lipid precursors and tracing isotope incorporation into plant lipid species using LC–MS/MS and compound-specific fatty acid analysis by GC–C–IRMS (Meier-Augenstein 2002). From a diagnostic perspective, the persistence of differentiation among nutritional groups after exclusion of chloroplast-associated fatty acids indicates that fatty acid profiling may complement stable isotope analysis when assessing potential fungal contributions to the carbon budgets of green plants, even if the observed profiles primarily reflect metabolic remodeling.

Several low-abundance fatty acids, including odd-chain and very-long-chain saturated compounds, also contributed to the fully mycoheterotrophic cluster. Odd-numbered straight-chain fatty acids occur in fungi and other microorganisms (Akinwole et al. 2014; Zhang et al. 2020) and may reflect microbial inputs associated with mycorrhizal tissues. This interpretation is consistent with evidence that fully mycoheterotrophic plants harbor distinctive bacterial associates in addition to mycorrhizal fungi (Herrera et al. 2020; Miller and Gans 2021). Very-long-chain saturated fatty acids, which are precursors of plant cuticular waxes, may instead reflect reinforced lipid barriers at symbiotic interfaces (Lewandowska et al. 2020; Batsale et al. 2021). However, cuticular composition varies among organs, and organ identity likely contributed to this signal (Guo et al. 2017). Organ-specific microbial communities may likewise have influenced some low-abundance compounds, further complicating their interpretation. These compounds are therefore best regarded as secondary descriptors rather than stand-alone biomarkers of full mycoheterotrophy.

Of the two focal green species, the rhizoctonia-associated orchid *Goodyera biflora* more closely resembled partially mycoheterotrophic taxa in the NMDS ordination, whereas *Trillium camschatcense*, a presumed autotrophic *Paris*-type AM plant with marked ^13^C enrichment, more closely resembled autotrophs. Pairwise PERMANOVA of the complete dataset supported this pattern: *T. camschatcense* did not differ significantly from autotrophs, and *G. biflora* did not differ significantly from partially mycoheterotrophic taxa. The mean individual 18:2n-6/18:3n-3 ratios showed the same pattern, with values of 0.43 in *T. camschatcense*, 1.23 in *G. biflora*, 0.42 in autotrophs, 0.94 in partially mycoheterotrophic taxa, and 12.53 in fully mycoheterotrophic taxa. These results are consistent with previous evidence against partial mycoheterotrophy in *T. camschatcense*, in which foliar ^13^C enrichment and AM colonization increased under greater light availability (Murata-Kato et al. 2022). In contrast, the similarity of *G. biflora* to partially mycoheterotrophic taxa is biologically plausible given the prevalence of partial mycoheterotrophy among rhizoctonia-associated orchids (Gebauer et al. 2016) and the occurrence of albino individuals in its close relative *G. velutina* (Suetsugu et al. 2019).

After exclusion of chloroplast-associated fatty acids, *G. biflora* did not differ significantly from fully mycoheterotrophic plants but differed significantly from partially mycoheterotrophic plants. Given the small sample sizes, the nonsignificant comparison does not establish equivalence between *G. biflora* and fully mycoheterotrophic taxa. Instead, excluding chloroplast-associated compounds appears to emphasize fatty acid variation unrelated to chloroplast abundance and may therefore distinguish less consistently between partial and full mycoheterotrophy.

Overall, fatty acid profiles captured biologically interpretable variation across the autotrophy–mycoheterotrophy continuum. Although no individual compound currently provides a definitive biomarker, a higher relative proportion of 18:2n-6 combined with a lower relative proportion of chloroplast-associated 18:3n-3 may represent a promising signal of increasing mycoheterotrophic dependence. Because stable isotope analysis does not always provide reliable evidence of partial mycoheterotrophy in green plants, fatty acid profiling may offer valuable complementary evidence for assessing fungal contributions to their carbon budgets. Whether the observed differences reflect metabolic remodeling associated with reduced photosynthetic function, incorporation of fungal-derived carbon into newly synthesized plant lipids, or direct retention of fungal lipids remains unresolved. Controlled culture experiments incorporating ^13^C labeling are needed to determine which fatty acids, if any, are transferred intact, remodeled, or synthesized from fungal-derived carbon. Matched comparisons of photosynthetic and nonphotosynthetic tissues within species would also help quantify organ effects and support the development of more reliable fatty acid indicators of partial mycoheterotrophy.

## Supplementary Information

Below is the link to the electronic supplementary material.

Supplementary Material 1 (XLSX 210 KB)

## Data Availability

Additional supporting information is available online in the Supporting Information section at the end of the article.
